# Surgical outcomes of percutaneous endoscopic lumbar discectomy in obese adolescents with lumbar disc herniation

**DOI:** 10.1186/s12891-023-06842-8

**Published:** 2023-09-06

**Authors:** Lianjun Qu, Yongli Wang, Fei Wang, Songou Zhang

**Affiliations:** 1 Department of Orthopedic Surgery, Shaoxing People’s Hospital, School of Medicine, Zhejiang University, Zhejiang Province, China; 2 Department of Orthopedic Surgery, Sunshine Union Hospital, Weifang, Shandong Province China; 3 Department of Anesthesiology, 80th Group Army Hospital, Weifang, Shandong Province China

**Keywords:** Percutaneous endoscopic lumbar discectomy, Lumbar disc herniation, Obese, Adolescent patients

## Abstract

In recent years, with improved living standards, adolescent obesity has been increasingly studied. The incidence of lumbar disc herniation (LDH) in obese adolescents is increasing yearly. No clinical studies have reported the use of percutaneous endoscopic lumbar discectomy (PELD) in obese adolescent lumbar disc herniation (ALDH) patients. This study evaluated the preliminary surgical outcomes of PELD in obese ALDH patients. Fifty-one ALDH patients underwent single-level PELD surgery between January 2014 and January 2020. Patients were divided into an obese group and a normal group. Patient characteristics and surgical variables were compared between the two groups. The VAS, ODI, and SF-36 scales were used preoperatively and postoperatively to evaluate the clinical efficacy. In this study, 19 patients were included in the obese group, and 28 were included in the normal group. There was no significant difference in age, sex, duration of low back pain, duration of leg pain, or operative level between the obese and normal groups preoperatively. The obese group had a longer operative time (OT) (101.9 ± 9.0 min vs. 84.3 ± 11.0 min, P < 0.001), more fluoroscopy exposures (41.0 ± 5.8 vs. 31.6 ± 7.0, P < 0.001) and a longer time to ambulation (29.9 ± 4.0 vs. 25.0 ± 2.9, p < 0.001) than the normal group. The groups did not significantly differ in complications. The VAS score for back and leg pain and the ODI and SF-36 score for functional status improved significantly postoperatively. The PELD procedure is a safe and feasible method for treating LDH in obese adolescents. Obese ALDH patients require a longer OT, more fluoroscopy exposures and a longer time to get out of bed than normal ALDH patients. However, PELD yields similar clinical outcomes in obese and normal ALDH patients.

## Introduction

Lumbar disc herniation (LDH) is the most common spinal degenerative disease, and it presents with persistent or recurrent radicular pain and positive root tension signs [1]. Although adolescent lumbar disc herniation (ALDH) is a rare cause of morbidity, it has shown an increasing tendency in recent years. However, while the pathogenesis is still unclear, it is associated with various factors, including trauma, genetic factors, dysplasia, and poor habits [2]. Surgical treatment is required when the standard conservative treatment fails or the condition worsens, seriously affecting the quality of life.

O’Connell was the first person to report the surgical treatment of ALDH in 1960 [3]. With the development of surgical technology and medical devices, surgical procedures for treating ALDH have included conventional open discectomy (COD), microdiscectomy (MD), microendoscopic discectomy (MED), and percutaneous endoscopic lumbar discectomy (PELD) in recent years. MD was developed with the potential to minimize soft tissue and muscle damage compared with the extensive surgical exposure required with COD. The potential advantages of the MD procedure compared with the COD procedure are less intraoperative blood loss, shorter hospitalization duration, lower risk of surgical site infection, earlier return to work, and no association with increased rates of perioperative complications [3, 4].

In 1997, Yeung et al. [5] developed the spinal percutaneous endoscopic surgical system, which allowed entry of the intervertebral disc through the safe area of Kambin and removal of nucleus pulposus tissue. Over the past 20 years, PELD has become increasingly popular in the treatment of lumbar degenerative disease in China. Liu et al. [6] suggested that percutaneous endoscopic transforaminal discectomy (PETD) can result in rapid recovery and better clinical outcomes after 2 years of follow-up than MED and MD. However, a meta-analysis showed that there was no firm evidence that minimally invasive lumbar discectomy procedures, such as MED and PELD, were superior to COD in terms of clinical outcomes, except for a shorter hospital stay and an earlier return to work [7]. Meanwhile, PELD has been widely used for treating ALDH patients and is considered a safe and effective surgical method [8, 9]. We have met many obese adolescent patients with LDH in our clinical practice. Although some studies have reported surgical results after PELD in ALDH patients, no study has reported the clinical outcomes of obese ALDH patients. Therefore, the purpose of this study was to analyze the clinical outcomes of obese ALDH patients undergoing PELD.

## Materials and methods

### Setting and patient population

One thousand and fifty consecutive patients who had undergone one-level PELD between January 2015 and January 2020 were assessed for the study (Sunshine Union Hospital, 80th Group Army Hospital, and Shaoxing People’s Hospital). The inclusion criteria were as follows: (1) a primary diagnosis of LDH; (2) 10–20 years of age at surgery; (3) persistent or recurrent low back pain and/or lower extremity radicular symptoms; (4) failure of conservative treatment for more than 6 months; and (5) last follow-up period one year after the operation. Patients with a previous history of lumbar spine surgery, mental illness, malignancy, instability, and surgical site infection were excluded. A body mass index (BMI) of ≥ 30 kg/m^2^ was considered to define obesity, and a BMI of 18.5–22.9 kg/m^2^ was considered to define normal body weight [10]. According to the above criteria, 47 patients were selected as the research subjects. We divided the subjects into an obese group and a normal group. The study was approved by the clinical research ethics committee of our hospital. All operations were performed by two single experienced spinal surgeons (FW and LJQ) using the same operative technique.

### Surgical technique

#### PTED

Patients were placed prone on a Jackson radiolucent spinal table. (1) Marking: The level of disc herniation, midline of the spinous process, and depth of the intervertebral foramen were identified by using the C-arm. We palpated the contour of the iliac crest and marked it on the body surface. According to the body habitus of the patients, the puncture point was marked 11–14 cm from the midline of the spinous process at L4-L5 and 12–16 cm from the midline at L5-S1. (2) Anesthesia and puncture: Remifentanil was administered 10 min before the operation. Then, the skin was infiltrated with 2 ml of 1% lidocaine, and an 18-G needle was introduced, anesthetizing the trajectory with 8–10 mL of 1% lidocaine. Under the guidance of C-arm fluoroscopy, the needle reached the ventral side of the vertex of the superior articular process (SAP), and then 2 mL of 1% lidocaine was locally injected. The guidewire was inserted into the puncture needle, which was removed, leaving the guidewire in place. Then, an 8-mm-long incision was made along the guidewire. Soft tissue expanders were used to create a path. (3) Foraminoplasty: The guidewire was removed, and a sharp Tom needle was passed through the tip of the SAP using a hammer. The tip of the Tom needle did not exceed the medial pedicle line on the anteroposterior X-ray view and did not enter the disc on the lateral view. Then, the sharp needle was replaced by a blunt needle tip. The guidewire was introduced through the blunt Tom needle, and the needle was then removed. A 4 mm cannulated drill was used to create a bone channel along the guidewire. Then, a 6 to 8 mm cannulated drill was used to enlarge the neuroforamen. (4) Placement of the working cannula and decompression of the nerve root: The drill was removed, and the 7 mm conical rod was replaced. The working channel was introduced over the conical rod, and the guidewire and conical rod were then removed. The tip of the working channel was located on the area of disc herniation. After adjusting the endoscopic system, the endoscope was inserted into the working channel, and part of the ligamentum flavum was removed. The dural sac, nerve root, and protrusion were identified. The nucleus pulposus herniation and the disc fragments around the nerve root were removed, and a radiofrequency electrode was used for hemostasis during the operation. Decompression was considered sufficient if the nerve showed pulsation with the heart rate and the amount of removed disc material matched the amount of disc herniation seen on the MRI. Then, the incision was closed with absorbable sutures.

#### Percutaneous interlaminar endoscopic discectomy (PIED)

Patients lay prone on a Jackson radiolucent spinal table under general anesthesia. On the anteroposterior lumbar spine X-ray, the shape of the interlaminar space at L5/S1 was marked on the surface of the skin. Then, the following steps were performed: (1) The spine needle was inserted vertically and slightly along the lateral edge of the interlaminar window on the symptomatic side. This and following procedures were monitored by anteroposterior and lateral radiography to ensure that the needle tip remained in the correct position and direction. (2) A guidewire was inserted along the spine needle, and a posterior transverse incision with a length of 7 mm was made at the entry point. (3) The expansion tube was used to gradually expand the soft tissue. (4) An extension tube was inserted until it was supported dorsally by the ligamentum flavum. (5) The working cannula was inserted over the extension tube. Then, the extension tube was withdrawn from the working cannula. The C-arm was repositioned to determine the position of the working cannula. (6) The endoscope was placed into the working cannula, and then the ligamentum flavum was dissected to enter the epidural space. After adequate exposure of the exiting nerve root and dural sacs, these structures were protected by rotating the working cannula. The herniated nucleus pulposus was removed with a grasper. (7) The endoscope and working channel were withdrawn after nerve root decompression was achieved. (8) A single stitch was placed to close the incision, which was then covered with a sterile dressing.

#### Clinical assessment

The operative time (OT), fluoroscopic exposures (FEs), hospital stay (HS), and time to ambulation (TTA) were recorded, and the SF-36, ODI and VAS questionnaires were used to assess the clinical outcomes of patients preoperatively, one day after surgery and then at one month, three months, six months, and one year after surgery. Complications were recorded during the surgery and follow-up period.

### Statistical analyses

Statistical analyses were performed utilizing SPSS statistical software v. SPSS 19.0 (SPSS, USA). Comparisons between the obese and normal groups were performed using independent sample t tests for continuous variables. Categorical variables were analyzed with a chi-squared test, and p < 0.05 was considered statistically significant.

## Results

Table 1 shows the demographic characteristics of the patients before surgery. The obese group comprised 12 (63.2%) males and 7 (36.8%) females, with a mean age of 16.5 ± 2.6 years. Their mean BMI was 37.2 ± 3.1 kg/m^2^. The control group comprised 15 (53.6%) males and 13 (46.4%) females, with a mean age of 15.7 ± 2.4 years. Their mean BMI was 20.2 ± 1.5 kg/m^2^. There was no significant difference in age, sex, duration of low back and leg pain or operative segment between the two groups before the operation.

**Table 1:** Comparison of preoperative parameters between the obese group and the normal group

Factor	Obese group	Normal group	P value
Patients (No.)	19	28	
Sex			0.561
Female (No.)	7	13	
Male (No.)	12	15	
Age (y)	16.5 ± 2.6	15.7 ± 2.4	0.244
BMI (kg/m2)	37.2 ± 3.1	20.2 ± 1.5	<0.001
Duration of LBP	12.6 ± 4.9	13.7 ± 4.5	0.387
Duration of leg pain	10.7 ± 3.7	11.3 ± 3.8	0.534
Spinal level			
L4-L5	8	12	
L5-S1	11	16	

LBP, low back pain. P < 0.05 was considered statistically significant

Table 2 shows the clinical and functional outcomes. In both groups, the VAS scores for back and leg pain and the ODI and SF-36 score for functional status significantly improved postoperatively. There was no significant difference in the VAS score or ODI between the groups preoperatively or one year postoperatively. Regarding the SF-36 score, there was a significant difference in the role-physical (RP) and bodily pain (BP) components between the two groups preoperatively and a significant difference in the BP and vitality (VT) components between the two groups at one year postoperatively.

**Table 2:** Comparison of the VAS score, ODI, and SF-36 score between the obese group and the normal group

Clinical outcome	Obese	Normal	p	Obese	Normal	p	Obese	Normal	p	Obese	Normal	p	Obese	Normal	p	Obese	Normal	P
	Preoperation			1 day post-op			1 month post-op			3 months post-op			6 months post-op			1 year post-op		
VAS score, back	3.1 ± 1.2	3.4 ± 1.1	0.446	2.0 ± 0.6	1.9 ± 0.7	0.591	1.5 ± 0.6	1.4 ± 0.6	0.404	1.2 ± 0.6	1.1 ± 0.7	0.632	1.1 ± 0.6	0.8 ± 0.6	0.097	0.7 ± 0.6	0.6 ± 0.6	0.575
VAS score, leg	6.5 ± 0.9	6.7 ± 1.0	0.514	1.8 ± 0.6	1.9 ± 0.8	0.764	1.8 ± 0.8	1.4 ± 0.6	**0.049**	1.4 ± 0.7	1.1 ± 0.7	0.117	1.1 ± 0.8	0.7 ± 0.5	**0.047**	0.5 ± 0.5	0.4 ± 0.5	0.377
ODI	58.2 ± 12.2	61.1 ± 12.7	0.423				18.0 ± 4.0	16.5 ± 4.4	0.220	13.4 ± 3.2	12.1 ± 3.3	0.174	10.0 ± 2.6	8.9 ± 2.2	0.122	6.4 ± 2.5	5.7 ± 2.1	0.321
SF-36 score																		
PF	44.7 ± 8.2	46.3 ± 8.8	0.174				56.8 ± 7.7	62.5 ± 9.2	**0.027**	68.5 ± 7.8	72.0 ± 7.5	0.127	72.4 ± 8.6	78.6 ± 7.1	0.087	74.2 ± 9.0	79.8 ± 7.5	**0.025**
RP	29.1 ± 22.7	32.1 ± 14.7	**0.018**				48.8 ± 20.6	57.1 ± 22.4	0.193	60.0 ± 18.8	71.4 ± 18.9	**0.044**	68.4 ± 16.3	78.6 ± 17.6	0.098	71.1 ± 17.2	79.5 ± 16.7	**0.102**
BP	36.6 ± 15.4	32.1 ± 14.7	**0.034**				49.8 ± 10.6	65.3 ± 14.7	**<0.001**	53.2 ± 10.1	69.5 ± 12.6	**<0.001**	70.2 ± 6.5	80.9 ± 6.6	0.400	69.6 ± 8.6	81.8 ± 7.0	**<0.001**
GH	58.4 ± 10.9	61.1 ± 15.3	0.200				60.9 ± 8.1	63.7 ± 10.0	0.303	62.9 ± 6.4	67.0 ± 8.1	0.069	65.7 ± 8.1	71.0 ± 7.7	0.659	67.5 ± 10.7	71.4 ± 11.4	0.244
VT	58.6 ± 11.4	61.7 ± 15.3	0.106				56.8 ± 9.4	65.5 ± 8.6	**0.002**	60.8 ± 9.2	71.4 ± 11.5	**0.001**	69.5 ± 5.7	78.9 ± 10.6	0.445	70.3 ± 7.5	80.6 ± 8.8	**<0.001**
SF	61.0 ± 14.6	56.4 ± 16.2	**0.032**				64.0 ± 13.4	73.8 ± 16.1	0.031	69.0 ± 11.7	80.5 ± 17.8	0.009	79.3 ± 11.0	84.5 ± 13.8	0.426	80.2 ± 18.7	83.9 ± 13.4	0.436
RE	51.2 ± 25.7	59.9 ± 24.0	**0.013**				75.2 ± 21.3	82.3 ± 19.1	0.230	76.8 ± 21.9	80.0 ± 16.4	0.570	79.3 ± 19.3	83.6 ± 18.8	0.769	80.8 ± 23.1	84.6 ± 21.2	0.562
MH	66.9 ± 12.6	67.6 ± 18.2	0.736				65.1 ± 10.1	73.6 ± 10.5	**0.008**	69.1 ± 8.2	77.7 ± 5.2	**<0.001**	71.8 ± 6.8	80.3 ± 7.4	0.166	74.1 ± 5.8	80.1 ± 5.9	**0.001**

VAS, Visual analog scale; ODI, Oswestry Disability Index; PF, physical functioning; RP, role-physical; BP, bodily pain; GH, general health; VT, vitality; SF, social functioning; RE, role-emotional, MH, mental health. P < 0.05 was considered statistically significant

Table 3 shows the perioperative outcomes. The obese group was associated with a longer OT (101.9 ± 9.0 min vs. 84.3 ± 11.0 min, P < 0.001) and more FEs (41.0 ± 5.8 vs. 31.6 ± 7.0, P < 0.001). Patients in the normal weight group were able to ambulate earlier (25 days) postoperatively than those in the obese group (29.9 days, p < 0.001). There was no significant difference in perioperative complications between the two groups.

**Table 3:** Comparison of intraoperative parameters and complications between the obese group and the normal group

Parameter	Obese group	Normal group	P
Operative time (min)	101.9 ± 9.0	84.3 ± 11.0	<0.001
Fluoroscopic exposures (n)	41.0 ± 5.8	31.6 ± 7.0	<0.001
Hospital stay (days)	3.3 ± 0.5	3.1 ± 0.5	0.070
Time to ambulation (days)	29.9 ± 4.0	25.0 ± 2.9	<0.001
Surgical approach			0.634
PTED	15	22	
PIED	4	6	
Anesthesia method			0.568
General anesthesia	5	8	
Local anesthesia	14	20	
Complications			1.000
Wound infection	0	0	
Dural tear	0	0	
Nerve root injury	0	0	
Major vessel injury	0	0	
Urinary injury	0	0	
Revision surgery	1	0	

PTED, percutaneous transforaminal endoscopic discectomy; PIED, percutaneous interlaminar endoscopic discectomy. P < 0.05 was considered statistically significant

Table 4 shows the perioperative outcomes. PTED was associated with more Fes (35.5 ± 8.0 vs. 30.1 ± 3.4, P = 0043). There was a significant difference in OT, HS, and TTA between the two groups. There was no significant difference in perioperative complications between the two groups.

**Table 4:** Comparison of perioperative outcomes between the PTED group and the PIED group

Parameter	PTED group	PIED group	P
Patients			0.634
Obese	15	4	
Normal	22	6	
Sex			0.011
Female (No.)	12	8	
Male (No.)	25	2	
Age (y)	16.3 ± 2.4	15.1 ± 2.8	0.190
Operative time (min)	92.0 ± 12.4	91.0 ± 13.9	0.822
Fluoroscopic exposures (n)	35.5 ± 8.0	30.1 ± 3.4	0.043
Hospital stay (days)	3.2 ± 0.5	2.9 ± 0.3	0.066
Time to ambulation (days)	27.5 ± 4.1	25.5 ± 4.2	0.178
Anesthesia method			<0.001
General anesthesia	3	10	
Local anesthesia	34	0	
Complications			1.000
Wound infection	0	0	
Dural tear	0	0	
Nerve root injury	0	0	
Major vessel injury	0	0	
Urinary injury	0	0	
Revision surgery	1	0	

PTED, percutaneous transforaminal endoscopic discectomy; PIED, percutaneous interlaminar endoscopic discectomy. P < 0.05 was considered statistically significant

## Complications

There were no complications, such as wound infections, dural tears, nerve root injuries, major vessel injuries or urinary injuries, in either group. One obese patient experienced recurrence of LDH within 1 month after the operation.

## Discussion

In recent years, with the improvement of people’s living standards and changes in diet structure, the incidence of obesity in China has increased markedly. Obesity has been implicated as a risk factor for LDH [11, 12]. Traditional open surgical treatments in obese patients require a large incision to allow adequate visualization at depth, which may result in greater injury of muscle and soft tissue, as well as increased bleeding and infection risks [13, 14]. Many spinal surgeons have used the PTED technique to treat obese patients with LDH in recent years. They suggest that PTED is a safe and generally effective minimally invasive technique for obese LDH patients [15, 16]. However, LDH is a rare cause of morbidity in obese adolescent patients. To our knowledge, there have been no studies on the application of the PTED technique for treating obese ALDH patients. Meanwhile, an increasing number of studies have suggested that PTED is a safe and efficient alternative to COD and MD for the treatment of ALDH [17, 18]. Therefore, the current study focused on investigating the efficiency of PTED in the treatment of obese adolescent patients with LDH.

The OT is usually one of the most concerning problems for patients. Chen et al. [19] reported that the mean duration of surgery was 97.2 min and the mean length of HS was 8.1 days in adult LDH patients with PTED. A recent meta-analysis comparing the transforaminal approach and interlaminar approach suggested that the average OT was 61.9–97.5 min and the average HS was 3.2–4.9 days in the PTED group [20]. Chen et al. [21] reported that the average OT in adolescents with LDH was 110 min longer than the 95 min reported in adults with LDH (P = 0.41). However, the surgical approaches included the transforaminal approach and interlaminar approach in both groups [21]. Bae et al. [22] reported that obese and normal patients spent a mean of 55 min and 51.8 min, respectively, in surgery. Meanwhile, the mean length of HS of obese and normal patients was 2.8 days and 3.3 days, respectively [22]. In our study, we found that the average OT in the obese adolescent group was 101 min, which was significantly longer than the 84.7 min in the normal group (P < 0.001), and the average HS (3.3 vs. 3.1; P = 0.070) was similar in the obese and normal groups. We observed different outcomes in each study. However, obese patients consistently spent more time in surgery. We also considered the differences between these studies mainly based on the following points. First, the subjects in each study had different conditions. Patients undergoing a more difficult surgery require a longer OT. Second, the PTED procedure has a steep learning curve, requiring a different set of cognitive, psychomotor and technical skills. Surgeons have different experiences, and repetition might lead to different clinical effects. Third, during the PTED procedure, it is more difficult to puncture and create the passage in obese patients than in normal patients because of the thick soft tissue layer in the former. Moreover, we believe that careful preoperative evaluation and good intraoperative fluoroscopy are very important for obtaining satisfactory surgical results in obese patients.

Many studies have encouraged patients to get out of bed early after PELD to recover their function and return to work early. Nie et al. [23] reported that the postoperative time in bed of 30 patients with L5–S1 disc herniation was 5.0 ± 1.1 h after PTED. Another study suggested that the mean postoperative in-bed time was 32.7 h in adult LDH patients after PTED [19]. In our study, we found that the average TTA (29.9 vs. 25.0 days; P < 0.001) was longer in the obese group than in the normal group. Considering the healing time of soft tissues such as the annulus fibrosus, our team recommends that patients stay in bed for at least 3 weeks after PELD. Of course, when eating or urinating, the patient can wear a waist brace and get out of bed. During other periods, patients mainly performed rehabilitation exercises in bed, mainly focusing on low back muscle exercises and limb strength exercises. Qin et al. [24] reported that the time to first ambulation after PELD was one of the crucial factors affecting recurrence after PELD. These results suggest that regardless of the lumbar protection method used, early ambulation will certainly subject the disc to a load too soon after lumbar surgery [24]. Therefore, in obese adolescent patients who get out of bed early after PELD, the load on the intervertebral disc could easily increase, resulting in an increase in the probability of postoperative recurrence. Therefore, we do not recommend that obese adolescent patients with LDH ambulate early after PELD.

Spinal surgeons often pay attention to their own radiation exposure in spinal surgery, and thus, patient radiation exposure is less frequently reported. In our study, we found that the frequency of X‑ray exposure was significantly increased in the obese patient group (41.0 ± 5.8) compared with the normal group (31.6 ± 7.0). Organs and tissues are more sensitive to the effects of radiation in childhood or adolescence than in adulthood [25]. Radiation exposure leads to a higher incidence of malignant tumors in young patients [26, 27]. However, the body surface markers of obese ALDH patients, especially their iliac crests, are not easy to palpate; thus, we increased the FEs to make improve the accuracy of the preoperative positioning of the surgical target. We are the first to report the fluoroscopic localization of the iliac crest in obese patients to allow appropriate insertion point selection and accurate channel placement. We believe that accurate preoperative body surface positioning is very important for obese ALDH patients undergoing PETD because of the difficulty of intraoperative puncture and channel adjustment. We used the PTED technique to treat all patients with LDH at the L4/L5 level. However, we selected two different endoscopy techniques (PTED and PIED) to treat patients with L5/S1 intervertebral disc herniation. For patients with a high iliac crest and narrow foramen at L5/S1, the PIED approach can be applied properly. In our study, we found that the number of X‑ray exposures was significantly increased in the PTED group (35.5 ± 8.0) compared with the PIED group (30.1 ± 3.4). Although obese ALDH patients have increased X-ray exposure, it is important to ensure safe and effective surgical outcomes. With additional experience in performing PELD in obese patients, we believe we can reduce the radiation exposure.

Many studies have reported that compared with other surgical procedures, PETD can achieve similar surgical results with less tissue damage [6, 19]. Bae et al. [22] also found that the clinical and functional outcomes of obese patients were similar to those of normal patients who had undergone PETD for LDH. In our study, we observed that both obese and normal patients showed significant improvements in the ODI and in back and leg pain VAS scores at 1 day post-operation. From 1 month after the operation, there was no significant difference between the 2 groups in the VAS score or ODI. Both the obese and normal groups showed greater improvement in the SF-36 score at 1 year post-operation.

Our study has several potential limitations that should be noted. First, the number of obese cases was relatively small, and the follow-up time was short. Second, due to the anatomic differences between L4/L5 and L5/S1, there were different surgical results during the implementation of PETD. This could also have led to outcome bias. Third, there was no imaging evaluation of the patients in our study. Nevertheless, a larger study with a longer follow-up is needed to further validate our clinical findings.

## Conclusions

The PELD procedure is a safe and effective method for the treatment of ALDH. PELD has a similar clinical outcome in the treatment of both obese and normal ALDH patients. However, obese ALDH patients require a longer OT, more FEs and a longer TTA than normal ALDH patients. Using the PELD procedure to treat obese ALDH patients requires careful preoperative evaluation, clear intraoperative fluoroscopy and experienced surgeons to improve the surgical effect and reduce the harm caused by surgery as much as possible.

## Data Availability

The datasets used and/or analyzed during the current study are available from the corresponding author upon reasonable request.
